# (Some) Cellular Mechanisms Influencing the Transcription of Human Endogenous Retrovirus, HERV-Fc1

**DOI:** 10.1371/journal.pone.0053895

**Published:** 2013-01-28

**Authors:** Magdalena Janina Laska, Kari Konstantin Nissen, Bjørn Andersen Nexø

**Affiliations:** Department of Biomedicine, Aarhus University, Aarhus, Denmark; Peking University Health Science Center, China

## Abstract

DNA methylation and histone acetylation are epigenetic modifications that act as regulators of gene expression. DNA methylation is considered an important mechanism for silencing of retroelements in the mammalian genome. However, the methylation of human endogenous retroviruses (HERVs) is not well investigated. The aim of this study was to investigate the transcriptional potential of HERV-Fc1 proviral 5′LTR in more detail, and examined the specific influence of CpG methylation on this LTR in number of cell lines. Specifically, the role of demethylating chemicals e.g. 5-aza-2′ deoxycytidine and Trichostatin-A, in inducing or reactivating expression of HERV-Fc1 specific sequences and the mechanisms were investigated. In our present study, 5-aza-dC is shown to be a powerful inducer of HERV-Fc1, and at the same time it strongly inhibits methylation of DNA. Treatment with this demethylating agent 5-aza-dC, results in significantly increased levels of HERV-Fc1 expression in cells previously not expressing HERV-Fc1, or with a very low expression level. The extent of expression of HERV-Fc1 RNAs precisely correlates with the apparent extent of demethylation of the related DNA sequences. In conclusion, the results suggest that inhibition of DNA methylation/histone deacetylase can interfere with gene silencing mechanisms affecting HERV-Fc1 expression in human cells.

## Introduction

Methylation of cytosine is one of the marks of transcriptionally inactive chromatin [1]. DNA methylation, taking place at cytosine residues located in CpG dinucleotides, involves the addition of a methyl group to the fifth carbon of the pyrimidinyl ring and the formation of 5-methylcytosine (mC). It is recognized that approximately 80% of CpG dinucleotides in the DNA of mammalian nonembryonic cells are methylated [2]. The distribution of methylated cytosine residues in eukaryotic DNA is nonrandom. Particularly, some regions are CpG-enriched yet practically devoid of methylation. These sequence stretches, termed ‘CpG islands’ (CGIs), are >500 bp on length and comprise ∼1% of total genomic DNA (e.g. the human genome contains more than 27 000 such islands, identified in the non-repetitive portions of the human genome) [3]. Also, 5-Methylcytosine occurs in repetitive sequences several-fold more frequently than in middle repetitive or unique sequences. Sequences that are comparatively rich in CpG dinucleotides include Alu, other SINE, L1 LINE (LINE-1) and certain satellite sequences [4]. This is consistent with the idea that hypermethylation is the default epigenetic state and serves in maintaining genome integrity. Methylation of CpG islands of promoter region correlates with inactivation of transcription of each gene, whereas demethylation of this region can induce activation of transcription [5]. In fact, a multitude of tissue-specific genes are repressed by promoter methylation. CpG methylation acts to suppress transcription in several ways [6]. Cytosine methylation can prevent the binding of some transcription factors, and DNA methylation can affect chromatin states indirectly through the recruitment of methyl-CpG-binding proteins (MBPs) [7]. The enzymes methylating DNA are known as DNA cytosine-5-methyltransferases (DNMTs). There are at least 4 independent methyltransferases (DNMT1, DNMT3a, DNMT3b and DNMT3L) participating in the process of DNA methylation related to maintenance of gene silencing. Thus, the importance of DNA methylation for gene activity is well documented.

The relationship between methylation and gene expression has been particularly well-studied for various retroviruses including Moloney murine leukemia virus, murine mammary tumor virus, avian sarcoma virus and others [8]. Epigenetic silencing is commonly observed after the transduction of therapeutic or reporter genes using retrovirus-based vectors and this event can occur at various incidences. The cause of retrovirus vector silencing has been attributed to *de novo* cytosine methylation of CpG sequences and subsequent histone deacetylation leading to chromatin condensation [9]. The epigenetic process that lead to retroviral silencing overlap extensively with those that regulate gene expression during embryonic development and cell differentiation [2]. Yoder JA, et.al suggested that an epigenetic silencing system initially evolved as a protective mechanism to silence transposable elements [9].

The methylation of human endogenous retroviruses (HERVs) is not well investigated. The few published studies suggested that proviruses and solitary LTRs are densely methylated under normal physiological circumstances, except in developing germ cells in the placenta [10]. *In vivo* HERV transcripts have been detected in numerous pathological situations and predominantly in the context of autoimmune/inflammatory diseases [11] [12]. Thus, HERV expression and transcriptional reactivation has been reported in some carcinomas with hypermethylated genomes, but at relatively low levels, e.g. HERV-H copy in gastrointestinal cancers, HERV-K family members in urothelial carcinomas and primary human testicular tumors, and HERV-W family in ovarian carcinomas [13] [14] [15]. In addition, treatment with DNA methylation inhibitors such as 5-aza-deoxycytidine increases the transcription of mRNA for HERV clone 4-1 and decreases transcription of mRNA for DNA methyltransferase 1 (DNMT1) in PBL from normal individuals [16]. CpG sites in various HERV-K (HML-2) proviral 5′LTRs are methylated at different levels in the cell line Tera-1. Here, methylation levels correlated with previously observed transcriptional activities of these proviruses [17]. Although more studies are required for all classes of endogenous retroelements, it is reasonable to conclude that their transcriptional activity is limited in somatic cells by DNA methylation.

HERV-Fc1, which is a part of the expanded HERV-H/F family, was identified with a full-length coding envelope gene in primates [18]. HERV-Fc1 is the most intact member of this entire group with almost complete *gag* and *pol* and intact *env* genes [18] [19] [20]. The *gag* ORF is terminated by two stop-codons, compared to the common single stop in exogenous retroviruses. The *pol* fragment is interrupted by a frameshift mutation and a premature stop-codon [19]. Transcripts from HERV-Fc1 *env* genes have been detected in different human tissues from testis, skin and trachea, indicating that its promoter is active [21]. We have reported that HERV-Fc1 is genetically and expressionally linked with an autoimmune disease Multiple Sclerosis [22] [23]. Still neither the methylation status of the HERV-Fc1 LTRs nor the methylation status of family related LTRs are known. Very little is known whether methylation can not only repress but also regulate HERV-Fc1 and other HERVs activity. Here, we investigated the transcriptional potential of HERV-Fc1 proviral 5′LTR in more detail, and examined the specific influence of CpG methylation on this LTR in number of cell lines. Specifically, the role of demethylating chemicals e.g. 5-aza-2′ deoxycytidine and Trichostatin-A, in inducing or reactivating expression of HERV-Fc1 specific sequences and the mechanisms were investigated.

Reports have demonstrated a synergistic effect of 5-aza-2′ deoxycytidine and Trichostatin-A in the re-expression of epigenetically silenced genes [24]. With an exception to HERV-K [25], currently it is not known how methylation patterns of human endogenous viruses are altered with 5-aza-2′ deoxycytidine. The present study was therefore designed to examine several factors that might influence the expression of retroviral sequences, using well established cell lines and primary cell strains. To elucidate whether hypermethylated retroelements might become reactivated upon treatment with demethylating agents, methylation levels of the predicted HERV-Fc1 promoter region were also quantified. Inhibition of protein synthesis was reported to increase the expression of endogenous retroviruses in mice [26]. Therefore, in a parallel line of experiments inhibitors of protein synthesis were investigated to see if they were able to increase the expression of human endogenous retrovirus like-sequences.

## Materials and Methods

### Cell Cultures

Human embryonic kidney (HEK) 293 cells were grown in Dulbecco's modified Eagle's medium (DMEM) supplemented with 10% fetal bovine serum (GIBCO, Grand Island, NY), 2 mM glutamine, 100 U/ml penicillin, 100 µg/ml streptomycin (Department of Biomedicine, DK) and used between passages 10–15. Jurkat cells and PBMCs were grown in RPMI medium supplemented with 10% fetal bovine serum 2 mM glutamine, 100 U/ml penicillin, 100 µg/ml streptomycin and used before passage 15. All cell lines were cultured in a humidified atmosphere in 95% air, 5% CO_2_ at 37°C. HEK 293 and Jurkat cells were purchased from ATCC (ATCC, Manassas, VA). PBMCs were obtained from blood samples of healthy volunteers using CPT™ tubes (BD Vacutainers®, BD Diagnostics, NJ, USA), and processed within 1 h according to the manufactures protocol. The Central Denmark Committee on Biomedical Research Ethics gave ethical approval for this protocol and all healthy volunteers gave their consent.

### Reagents and Antibodies

The following antibodies were used in this study: Anti-HERV-Fc1 Gag antibody (custom-made Apronex Biotechnologies), anti-tubulin (catalogue #4074 Abcam, Cambridge United Kingdom), anti- acetyl histone H3 and H4 (catalogue #06-598 Millipore, USA), and anti-rabbit goat secondary antibody (catalogue #sc-2004, Santa Cruz, USA). Trichostatin-A, 5-aza-2′ deoxycytidine, cycloheximide, MG-132 (Z-Leu-Leu-Leu-CHO) were purchased from Sigma (Sigma, St Louis, MO, USA).

### Cell viability assay

A Trypan blue, 0,4% solution (Sigma, St Louis, MO, USA) exclusion assay was carried out after treating cells with different concentrations of 5-aza-dC and/or TSA for different time periods. Viability of control mock-treated cells was set as 100%.

### Generation of HERV-Fc1 Gag antiserum

For antiserum production, a sequence encoding the N-terminal 257aa of HERV-Fc1 Gag was inserted in a pTHUB-vector (pBR328 basic vector with T7 promoter and N-teminal 6×His-ubiquitin-fusion sequence). The fusion protein was expressed in *E. coli* and used for immunization of rabbits under contract with Apronex Biotechnologies, CZ. Preimmune serum was collected before peptide immunization. The rabbit was immunized with 350 µg antigen in 50 mM TrisHCl, 5 mM 2-Me and 0.5% Triton ×100 and boosted 3 times and after the final boost, peripheral blood was collected for subsequent measuring of anti-peptide antibodies. The specificity and cross-reactivity of the anti-HERV-Fc1 Gag antiserum was analyzed in enzyme-linked immunosorbent assay (ELISA).

### Transient expression of HERV-Fc1 Gag in studied cell lines

In experiments involving overexpression of HERV-Fc1 Gag protein, pcDNA3.1 (+)/mycHis A Fc1 Gag or pcDNA3.1 (+)/mycHis A vectors were transiently transfected into the indicated cells by calcium phosphate transfection method as described by Flemington laboratory. Transfection efficiency was determined simultaneously by transfecting green fluorescence protein expressing plasmid pEGFP1 (Clontech, CA, USA). pEGFP1 vector or pcDNA3.1 (+)/mycHis empty vector were used for mock transfections as well as an loading control for comparison of protein expression. All expression vectors used in this study were driven by the CMV immediate early promoter.

### Western Blot

Whole-cell extracts were prepared after lysis in NP-40 lysis buffer (10 mM Tris-HCl pH 7.4, 137 mM NaCl, 10% v/v glycerol, 1% v/v Nonidet P-40) containing a proteasome inhibitor cocktail (Roche diagnostics, DK). Cell debris were removed by centrifugation at 10,000 *g* for 25 min at 4°C and protein concentration determined by BCA assay (Pierce, VWR/Bie & Berntsen, Herlev, DK). Equal amounts of protein (20 µg/sample) were separated by SDS-polyacrylamide gel electrophoresis, transferred to nitrocellulose membranes, and incubated with specific antibodies, followed by incubation with HRP-conjugated secondary antibody. Immunoblots were developed by enhanced chemiluminescence using proprietary reagents (Millipore, Copenhagen, DK).

### Proteasome inhibition assay

HEK 293 cells or Jurkat cells were cultured as described above. To inhibit proteasome activity, MG-132 (10 µM) was added to conditional medium and the cells were incubated for the indicated time periods. The cells were then harvested in NP-40 lysis buffer by scraping on ice, transferred into micro tubes, and centrifuged at 12,000 *g* for 25 min. The supernatants were used as protein extracts.

### RNA isolation, cDNA synthesis and real-time Q-PCR

Total RNA was extracted from cultured cells using TriReagent (Sigma, St Louis, MO, USA) according to the manufacturer's instructions. 1 µg total RNA was used for cDNA synthesis using iScript™ cDNA synthesis kit (Bio-Rad, CA, USA) according to the instructions of the manufacturer. Real-time Q-PCR analysis was performed using a LightCycler 480 cycler (Roche Diagnostics, DK). 2 µl of cDNA (from a total 20 µl reaction volume) was used in a 20 µl reaction. The real-time Q-PCR reactions contained 10 µl SybrGreen 2× Master Mix (Roche Diagnostics, DK), 2 µl forward primer (5 pmol/µl), 2 µl reverse primer (5 pmol/µl) and 4 µl water. After initial denaturation at 95°C for 10 min, PCR amplifications were performed for 45 cycles. The primer sequences for HERV-Fc1 extracellular *gag* RNA Fc1 1F: TGCAGAAGACAAGGCAATG Fc1 1R: AGTGTTCCCTTGGACAGGTG, oligonucleotide primers used to detect the expression of the coding envelope were Fc1 *env* 1F: TCCGATGGAGGTTCTACCTG Fc1 1R: GGCGATAGGTGTGTTGGAGT and for ß-actin 1F: TTCAAC ACCCCA GCCATG T 1R: TGTGGT ACGACC AGAGGC ATA C. These primers were expected to amplify both unspliced and spliced *env* transcripts. The crossing point (CP) for each transcript was measured and defined at constant fluorescence level in Light Cycler 480 software (Roche Diagnostics, DK). The mRNA levels for the test gene were normalized to the ß-actin value and relative quantification was determined using the ΔCt model presented by PE Applied Biosystems (Perkin Elmer, Foster City, CA). For the purpose of calculating fold changes in expression, genes with no detectable expression were assigned a C_t_ value of 40. Standard deviations were calculated and a T-test was employed to compare expression levels in drug treated cells against untreated cells. *P*-values less than 0.05 were considered statistically significant.

### Genomic DNA extraction

Total genomic DNA was extracted from cells using a standard proteinase K (Roche, Mannhein, Germany) digestion (overnight at 55°C), followed by phenol/chloroform extraction and DNA precipitation using ethanol. DNA was recovered by centrifugation, dissolved in TE (Tris 10 mM, EDTA 1 mM, pH 8), and stored at −20°C.

### EpiTect Methyl DNA Methylation qPCR Primer Assays

For methylation analysis of the HERV-Fc1 locus in the studied cell lines, the EpiTect Methyl qPCR Assay System (SABiosciences, Frederick, MD) was employed. EpiTect Methyl qPCR Assay is a simple and reliable method for quickly detecting the DNA methylation status of the CpG islands associated with individual genes. The system is capable of detecting DNA methylation with predesigned primers but without the bisulfite conversion. The EpiTect Methyl qPCR primer assay corresponds to one distinct CpG island in the promoter region of the gene. For the HERV-Fc1 locus we included sequences comprising 5′LTR region and part of the *gag* gene region. EpiTect Methyl qPCR primers were first designed by a rigorously optimized computer algorithm that accounts for the GC-rich sequences in genomic DNA and particularly in CpG islands. The design algorithm also ensures that every amplicon contains sufficient restriction sites for both methyl-sensitive and methyl-dependent enzymes to maximize methylation detection sensitivity. DNA methylation-sensitive restriction enzymes and/or methylation-dependent restriction enzymes were used to obtain the products containing hypermethylated DNA sequences or unmethylated DNA sequences, respectively. We digested 250 ng of DNA from HEK 293 and/or Jurkat cells with Methyl-Profiler™ DNA Methylation Enzyme Kit (SABiosciences) at 37°C for 6 h. Subsequently the enzymes were inactivated at 65°C for 20 min. The remaining DNA after digestion is quantified by real-time PCR using primers that flank the region of interest within the 5′LTR-*gag* sequence of the HERV-Fc1. The HERV-Fc1 locus CpG products were amplified using Light Cycler 480 machine and were analyzed based on the C_t_ values. The PCR cycling protocol was as follow: 1 cycle (95°C for 10 min), 3 cycles (99°C for 30 sec; 72°C for 1 min), and 40 cycles (97°C for 15 sec; 72°C for 1 min). Using the SABiosciences company's Excel-Based Data Analysis Template, we have obtained the percentage of hypermethylated DNA. The relative quantities of differentially methylated DNA (specifically hypermethylated, intermediately methylated, and unmethylated DNA) were determined by comparing the amount in each digest with that of a mock digest. The fraction of DNA in each digest was calculated by normalizing the DNA amount to the amount of digestible DNA. The amount of digestible DNA is equal to the total amount of DNA (determined from the mock digest) minus the amount of DNA resistant to DNA digestion (determined from the double digest).The differences in C_t_ values between the double and mock digest (ΔC_t_ [M_sd_-M_o_]>2) represents the analytical window (W) of the assay. In all experiments the W was >2, which means that more than 75% of all DNA molecules in the samples were digested; hence the results are reliable and meaningful. Methylation-sensitive or methylation-dependent digest C_t_ values within one cycle of the mock digest cannot be reliably used to calculate the percentage of either respective methylated DNA fraction. In these situations, the digest with the greatest difference in C_t_ value from the mock digest was used to calculate its methylated DNA fraction, whether unmethylated or hypermethylated. The opposite fraction was instead calculated as one minus the determined fraction. The amount of intermediately methylated DNA was then assumed to be negligible.

## Results

### Dose response of 5-aza-2′deoxycytidine and Trichostatin-A induced HERV-Fc1 *gag* and *env* mRNA expression

DNA methylation and histone acetylation can be modified with the chemical agents 5-aza-2′-deoxycitidine (5-aza-dC) and Trichostatin-A (TSA) respectively. The drug 5-aza-dC is a nucleoside analog which is incorporated into cellular DNA and irreversibly inhibits DNA methyltransferase apparently by forming a covalent complex in the catalytic site of the enzyme [27]. This depletion of methyltransferase in the cell results in passive demethylation, which is known to reactivate epigenetically silenced genes. The agent was originally developed as a cancer chemotherapeutic agent and is a powerful inducer of genes silenced by DNA methylation. It is used clinically to treat leukemia and the myelodysplastic syndromes and has been used experimentally to treat ß-thalassemia and sickle cell disease [28]. Another agent which affects the epigenetic status of genes is TSA. TSA is a potent, specific inhibitor of histone deacetylase activity. This drug leads to gene activation through hyperacetylation of histones.

**Figure 1:**
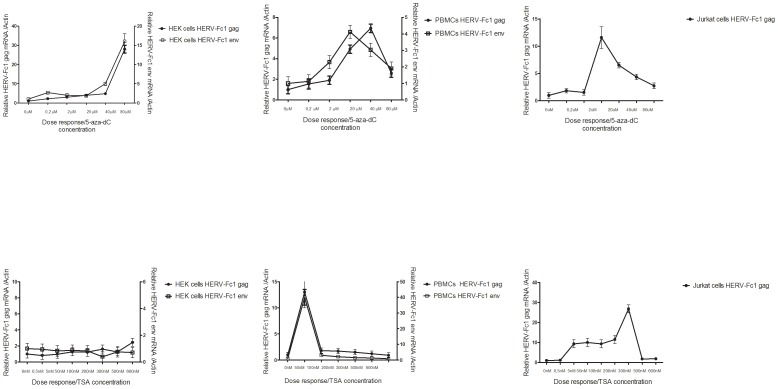
Dose response of 5-aza-2′deoxycytidine and Trichostatin-A induced HERV-Fc1 *gag* and *env* mRNA expression. Effect of different doses of 5-aza-dC (upper row) or TSA (bottom row) on the HERV-Fc1 *gag* and *env* extracellular mRNAs expressions. Cells were cultured in medium (untreated control) or in medium containing indicated drug at the indicated concentrations. After 24 h cells were harvested, RNA was extracted and used for HERV-Fc1 *gag* and *env* quantification by real-time Q-PCR. Relative changes of HERV-Fc1 *gag* and *env* expression in HEK 293, PBMCs and Jurkat cells. Q-PCR results shown are the means of two experiments each performed in triplicates. The results are relative to the untreated control cells. Errors bars represent ± SD. Y-axis relative HERV-Fc1 mRNA expression levels.

To demonstrate that CpG methylation is an epigenetic silencer of HERV-Fc1 transcription in HEK 293, PBMCs, and Jurkat cells, the cells were treated with the demethylating agent 5-aza-dC alone or in combination with TSA. Combined treatment was performed on the cells to observe whether histone acetylation would influence the process of DNA demethylation. After treatment, changes in HERV-Fc1 expression were measured by quantitative real-time PCR using primers specific for *gag* and *env* respectively. Based on a literature search, we chose an initial 5-aza-dC drug concentration ranging from 2 µM–80 µM. At these concentrations of 5-aza-dC, a cell viability of 70–80% was observed, as assessed using trypan blue staining. At the higher doses tested 5-aza-dC showed cytotoxicity, thereby severely affecting cellular viability and subsequently impairing HERV-Fc1 expression (data not shown). Figure 1 shows that after 24 h of incubation with 5-aza-dC, treatment altered the expression of HERV-Fc1 *gag* and *env* specific mRNA sequences in all tested cells. All concentrations of 5-aza-dC produced higher peak levels of *gag* and *env* HERV-Fc1 mRNA as compared to untreated control cells. Figure 1 shows that a dose of 40–80 µM provided a near-maximal increase in HERV-Fc1 gene expression in the HEK 293 cells and PBMCs and even the lowest dose tested stimulated significantly HERV-Fc1 *gag* and *env* mRNA accumulation. As for the Jurkat cells, Figure 1 shows that lower concentration of 5-aza-dC accelerated expression of HERV-Fc1 *gag* specific mRNA sequences, with cell growth rates declining significantly at higher doses. Peak expression was obtained within 24 h at concentration as low as 2 µM–20 µM, with sustained cell viability ranging between 70–80%, whereas with higher doses the curve fell abruptly. We were not able to detect any HERV-Fc1 *env* mRNA after 24 h incubation in this cell line, longer incubation time was required. Significant differences were observed in the resulting levels of HERV-Fc1 *gag* mRNA expression between cell lines/strains stimulated with 5-aza-dC, with HEK 293 and Jurkat cells showing the highest *gag* mRNA expression.

In order to study the influence of histone acetylation on the transcriptional activity of HERV-Fc1, cells were treated with the histone deacetylase inhibitor TSA. In HEK 293 cells, TSA failed to reactivate expression of HERV-Fc1 *gag* and *env* mRNAs to a level significantly different from untreated control (Figure 1). TSA increased the expression of HERV-Fc1 transcribed genes in PBMCs and Jurkat cells (Figure 1). In PBMCs, TSA treatment at lower concentration, ranging from 50 nM–100 nM elevated the expression of HERV-Fc1 *gag* and *env* mRNAs by approximately 14 fold, as compared to untreated control cells. Our results also confirm TSA-mediated induction of HERV-Fc1 *gag* gene transcription in Jurkat cells, though at higher doses. An average 27-fold increase was observed at 400 nM. Similar to 5-Aza-dC treatment of Jurkat cells, longer incubation time was required to detect alterations in HERV-Fc1 *env* mRNA expression level.

### 5-aza-dC and/or TSA stimulates HERV-Fc1 expression in human cells

**Figure 2:**
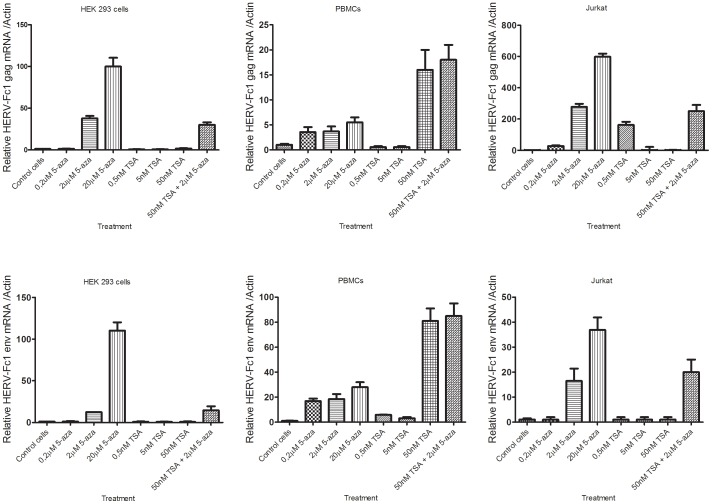
5-aza-dC and/or TSA stimulated HERV-Fc1 expression in human cells. Changes of HERV-Fc1 expression in HEK 293 (A), PBMCs (B), and Jurkat cells (C) after treatment with epigenetic agents. Cells were cultured in medium (untreated control) or in medium containing indicated drug at the indicated concentrations (5-aza-dC, TSA or 5-aza-dC and TSA). After 3 days cells were harvested, RNA extracted and used for HERV-Fc1 *gag* and *env* real-time Q-PCR analyses. Graph bars represent relative changes of HERV-Fc1 *gag* and *env* extracellular mRNA. Q-PCR results shown are the means of two experiments each performed in triplicates. The results are relative to the untreated control cells. Errors bars represent ± SD. Y-axis relative HERV-Fc1 mRNA expression levels.

To determine whether 5-aza-dC and/or TSA treatment might have a stronger effect on HERV-Fc1 transcription if applied over a longer time course, we performed a separate experiment, in which cells were exposed to agents for 3 days, replenishing the drug containing medium every 12 h. Briefly, four study treatments were established. The groups consisted of treatment with TSA, 5-aza-dC, TSA and 5-aza-dC, or culture medium alone. The cell lines were treated with varying concentrations of TSA and 5-aza-dC, which were previously empirically determined for optimal HERV-Fc1 *gag* and *env* gene up-regulation along with minimal cell death and cytotoxicity. The effects of the longer incubation period were as expected, with HERV-Fc1 *gag* and *env* mRNAs levels being significantly higher at 3 days that they were at 24 h (Figure 2). A very strong increase of HERV-Fc1 *env* expressions were detected in Jurkat cells (>600-fold upregulation) (Figure 2c). However, this very high induction is related to very low levels in the untreated cells, rather than a very high level in the induced cells. The optimal up-regulation of both *gag* and *env* mRNAs expression, along with minimal effect on cell viability, was induced by concentrations of 2 µM and 20 µM 5-aza-dC for all cell lines, and 50 nM TSA (for PBMCs) and 0,5 nM TSA (for Jurkat cells), respectively. These concentrations were used throughout the remainder of the study. The HEK 293 cell line overall showed no increase in HERV-Fc1 *gag* and *env* mRNAs expression when treated with TSA alone (Figure 2a). PBMCs had a significant increase in HERV-Fc1 expression (>15 fold over control) after TSA treatment (Figure 2b). In PBMCs, TSA was the most effective agent for HERV-Fc1 gene up-regulation, while in HEK 293 and Jurkat cells, 5-aza-dC was the most powerful drug to activate HERV-Fc1 transcription. Co-treatment of HEK 293 cells, PBMCs and Jurkat cells with 5-aza-dC and TSA did not show a synergistic effect on the expression of HERV-Fc1 *gag* and *env* mRNAs. In fact, combination of TSA and 5-aza-dC treatment did not extend the degree of HERV-Fc1 expression seen with each drug alone. Thus there was no influence of histone acetylation on the re-methylation process in these cells. The lack of TSA response in HEK 293 cells indicates that transcriptional regulation by histone acetylation may not be a key regulator of HERV-Fc1 expression in HEK 293 cells.

### HERV-Fc1 Gag protein synthesis in 5-aza-dC treated cells

To test the hypothesis that an increase in HERV-Fc1 transcription might even result in detectable HERV-Fc1 Gag protein expression, we measured Gag expression by Western Blot (Figure S1). We checked whether these levels of RNA were sufficient to yield detectable HERV-Fc1 Gag protein levels in all cells studied. Cells transfected with a HERV-Fc1 Gag expression vector (Fc1 Gag pcDNA3.1 (+)/mycHis A) were used as a positive control. We were able to detect weak HERV-Fc1 Gag protein expression only in HEK 293 cells treated with 20 µM 5-aza-dC. None of the TSA/5-aza-dC treatments led to detectable HERV-Fc1 Gag protein production in PBMCs and Jurkat cells. Those data are in accordance with previous reports showing that treatment with 5-aza-dC appears to reactivate many genes at the transcriptional level, without a corresponding reactivation at the protein level. Furthermore other epigenetic mechanisms, besides methylation, may account for the regulation of HERV-Fc1 Gag protein expression in those cells. However we cannot exclude the possibility that the antibody's sensitivity is below the level allowing for detection of endogenous Gag proteins by Western Blot technique.

### 5-aza-dC and TSA downregulate DNA methyltransferase 1 (DNMT1)

**Figure 3:**
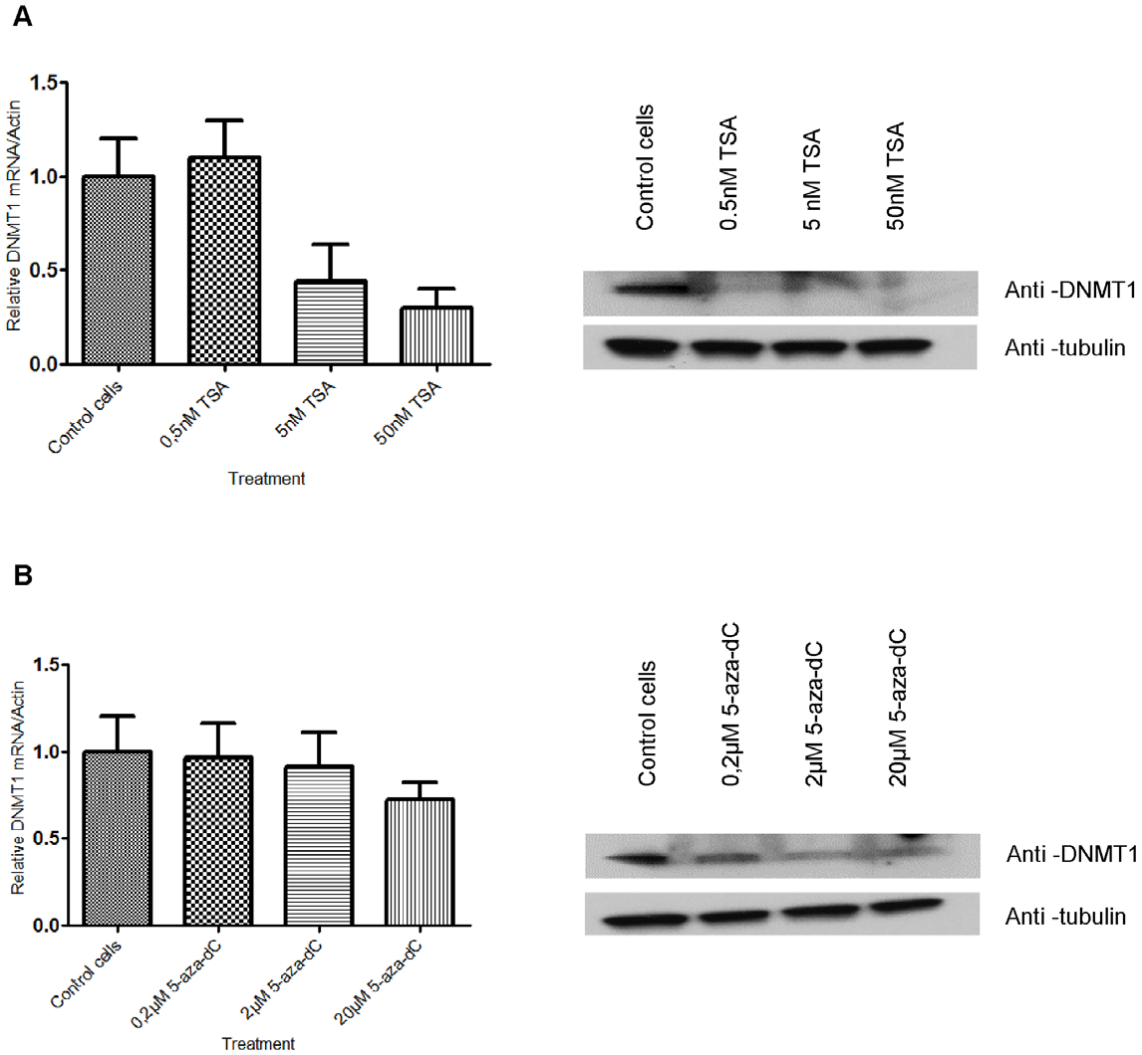
5-aza-dC and TSA downregulation of DNA methyltransferase 1 (DNMT1). (A) TSA down-regulate DNMT1 in DNMT1 mRNA and protein in Jurkat cells. Jurkat cells were incubated either without or in the presence of TSA at the indicated concentrations for 24 h. After incubation, total RNA and total protein extract were isolated. The DNMT1 transcript levels were determined by real-time Q-PCR analysis of cDNA. The results were standardized with ß-actin cDNA level. Each sample was determined in triplicate and results represent means ±SD from two experiments. The results are relative to the untreated control cells. Western blotting with anti-DNMT1 antibody. Anti-tubulin antibody was used as a loading control. (B) 5-aza-dC regulated expression of DNMT1 mRNA and protein in Jurkat cells. Jurkat cells were incubated either without or in the presence of 5-aza-dC at the indicated concentrations for 24 h. After incubation, total RNA and total protein extract were isolated. The DNMT1 transcript levels were determined by real-time Q-PCR analysis of cDNA. The results were standardized with ß-actin cDNA level. Each sample was determined in triplicate and results represent means ±SD from two experiments. The results are relative to the untreated control cells. Western blotting with anti-DNMT1 antibody. Anti-tubulin antibody was used as a loading control.

To demonstrate that DNMT1 degradation contributes to the demethylation induced by 5-aza-dC and to further examine the correlation with transcriptional activation of HERV-Fc1, we measured mRNA and protein level of DNMT1 after 5-aza-dC exposure. Additionally, we decided to evaluate the TSA effect on DNMT1 mRNA and protein expression. Protein levels of the class I deacetylases HDAC3, class IIA HDAC4 and the maintenance of DNMT1 were also surveyed via Western Blot using commercial antibodies. We moreover investigated the sensitivity of cells to DNMT1 mRNA degradation by Q-PCR. The cells were treated with 5-aza-dC for 72 h. Q-PCR revealed a minor down-regulation in DNMT1 mRNA expression levels (Figure 3a). Loss of DNMT1 protein was observed (Figure 3a) but to the negligible extent. The experiments revealed that higher concentration of 5-aza-dC decreased DNMT1 protein level at 72 h. The results were consistent with earlier reports showing that 5-aza-dC can trap DNMT1 by covalently linking the enzyme to DNA or lead to proteasome degradation of the DNMT1 protein [29]. Our preliminary results indicate that 5-aza-dC mediated enhancement of transcription of HERV-Fc1 *gag* and *env* sequences is synchronous to 5-aza-dC induced downregulation of DNMT1. Additionally, we observed that TSA resulted in a 2, 5- to 3-fold reduction of DNMT1 mRNA after 72 h of treatment of Jurkat T cells when the results were standardized against ß-actin transcript (Figure 3b). Similar results were obtained for other cell lines tested (data not shown). Alteration in DNMT1 protein content was determined by Western Blots. These experiments revealed that increasing doses of TSA progressively decreased DNMT1 protein level (Figure 3b). Next, we evaluated effects on the biological targets of HDACs. Inhibition of class I and class IIA HDACs with TSA led to a robust and global hyperacetylation detectable with H3ac and H4ac antibodies (Figure S2). Finally, interfering with DNA methylation by 5-aza-dC treatment moderately enhanced global H3 and H4 acetylation levels (data not shown).

### Association between HERV-Fc1 5′LTR methylation and transcriptional activity

**Figure 4:**
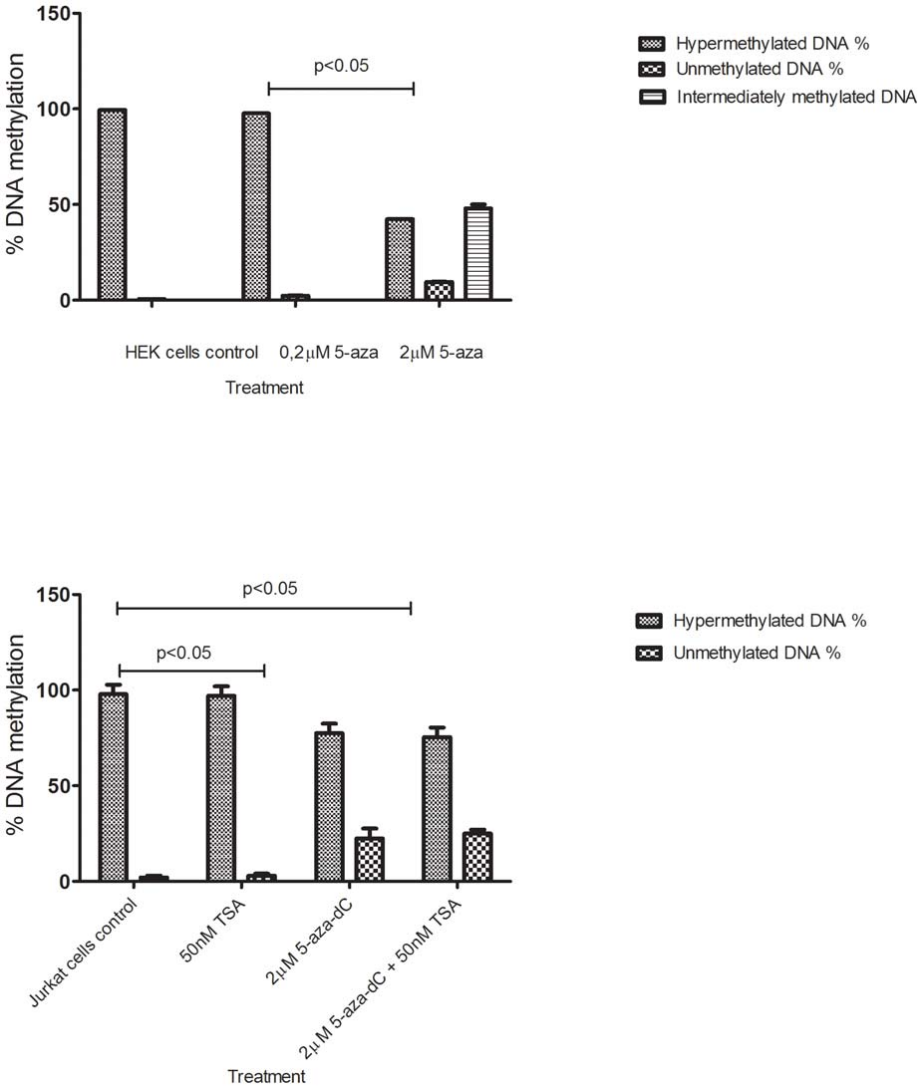
DNA methylation of HERV-Fc1 5′LTR-*gag* region. DNA methylation status of HERV-Fc1 5′LTR-*gag* region in (A) HEK 293 and (B) Jurkat cells treated with 5-aza-dC, and/or TSA for 24 h. Drug concentrations are indicated. Percentage of the hypermethylated, unmethylated, and intermediately methylated DNA is shown. Column bars show the median values and ±S.D. P-values show statistical differences (<0.05) between the different groups. P values were calculated using the non-parametric Mann-Whitney *U*-tests. Y-axis show percentage of DNA methylation.

We compared the level of methylation within the HERV-Fc1 regulatory region in human cultured cells. Using the EpiTect Methyl technology, we assayed the CpG methylation level within the 5′LTR–*gag* region of the HERV-Fc1 provirus. For all samples the analytical window (W) values were >2 (average 11,75 for an assay) and the R values representing the refractory DNA values were <25% (average 2,9%). Whilst all studied cell lines displayed hypermethylation, there were some differences in HERV-Fc1 methylation levels indicating these cells exhibit a unique methylation profile. The HEK 293 cell line displayed higher methylation level (99,49%) within 5′LTR-*gag* region of HERV-Fc1 compared to the Jurkat cell line which had a total methylated cytosine content of 97,40%. Following treatment of cells with 5-aza-dC over 24 hours, there was a substantial decrease of genomic HERV-Fc1 5′LTR-*gag* DNA methylation. The decrease in methylation in the HEK 293 cell line was greater than 55%, whilst the decrease in Jurkat cells was approximately 30% (Figure 4a and b). Thus, all treated cells had significantly lower levels of methylation compared to untreated cells (p<0.01). Combined TSA and 5-aza-dC treatment was performed on the Jurkat cells to observe whether histone acetylation would influence the process of DNA demethylation. Measurement of genomic methyl-cytosine levels revealed no synergistic effect of combined TSA and 5-aza-dC treatment on the demethylation and no influence of acetylated histones on the remethylation process in those cells. Comparison of the mRNA expression of HERV-Fc1 *gag* with the respective DNA methylation shows an inverse relationship between expression levels and % methylation. As seen in Figure 1, almost no or very low HERV-Fc1 *gag* expression is seen in the untreated HEK 293, and Jurkat cells. This corresponds to a methylation level of near 100% in both cell lines. Culturing per se did not change methylation levels and did not induce HERV-Fc1 *gag* mRNA expression. Presence of 5-aza-dC resulted in a considerable loss of methylation, as expected and induced HERV-Fc1 *gag* expression. In all experiments the enzyme digestion efficiency was carefully controlled.

We further investigated the relationships between gene transcription and the remethylation of CpG islands within HERV-Fc1 LTR-*gag* region. The remethylation kinetics of the 5′LTR –*gag* HERV-Fc1 region was examined because CpG islands within this region are hypermethylated in human cells, and as shown (Figure 4a and b) they become significantly demethylated by 5-aza-dC in vitro. Levels of demethylation were measured 72 h after drug addition. Levels of remethylation were measured beginning removal of 5-aza-dC at day 3 (day 1 drug free culturing).

**Figure 5:**
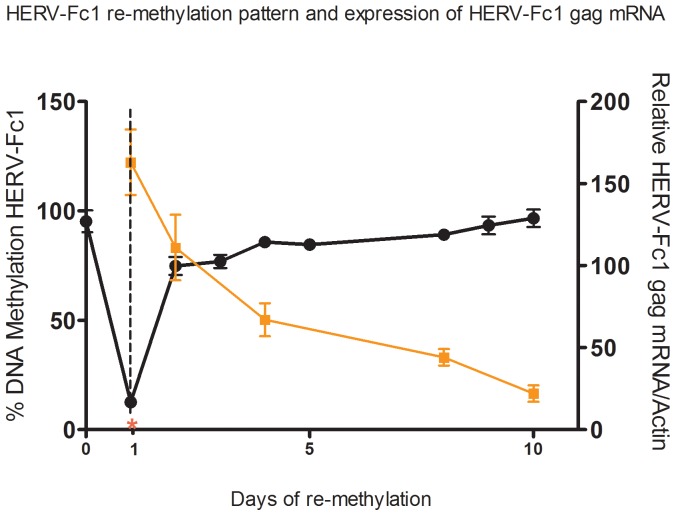
Association between HERV-Fc1 5′LTR methylation and transcriptional activity. Global remethylation in Jurkat cells and comparison of DNA methylation status and HERV-Fc1 mRNA expression. Jurkat cells exhibited demethylation after 5-aza-dC treatment for 72 h. Remethylation occurred over the 10 days of drug free growth. The red start and the dotted line indicate the time point when 5-aza-dC was removed from the culture and cells were subsequently cultured in drug-free medium. RNA and DNA were extracted simultaneously from the same sample of Jurkat cells before the culture, after 72 h treatment with 5-aza-dC, and during the 10 day period of drug-free culture. Comparison of the mRNA expression of HERV-Fc1 *gag* with its respective DNA methylation shows an inverse relationship between expression levels and % methylation. The error bars represents ±SD.

The rate of remethylation of HERV-Fc1 5′LTR-*gag* genomic region in cells was determined by EpiTect Methyl technology as a function of the lowest level of methylation after treatment (Figure 5). The HERV-Fc1 5′LTR-*gag* locus showed significant remethylation between 1 and 10 days of drug free treatment. By the tenth day of drug free growth, global HERV-Fc1 methylation levels approached those observed prior to drug treatment (Figure 5). Next, the level of expression of reactivated HERV-Fc1 *gag* gene in the days following removal of 5-aza-dC was investigated. Figure 5 shows that HERV-Fc1 *gag* was activated and highly expressed 10 days after 5-aza-dC removal. These data are consistent with the hypothesis that transcription does not block remethylation of endogenous genes, but that other factors than overall methylation are important for expression. For instance, HERV-Fc1 transcription may be associated with the remethylation of specific CpG islands within the transcribed regions of the virus.

### Cycloheximide increases endogenous HERV-Fc1 retroviral RNA levels in human cells

**Figure 6:**
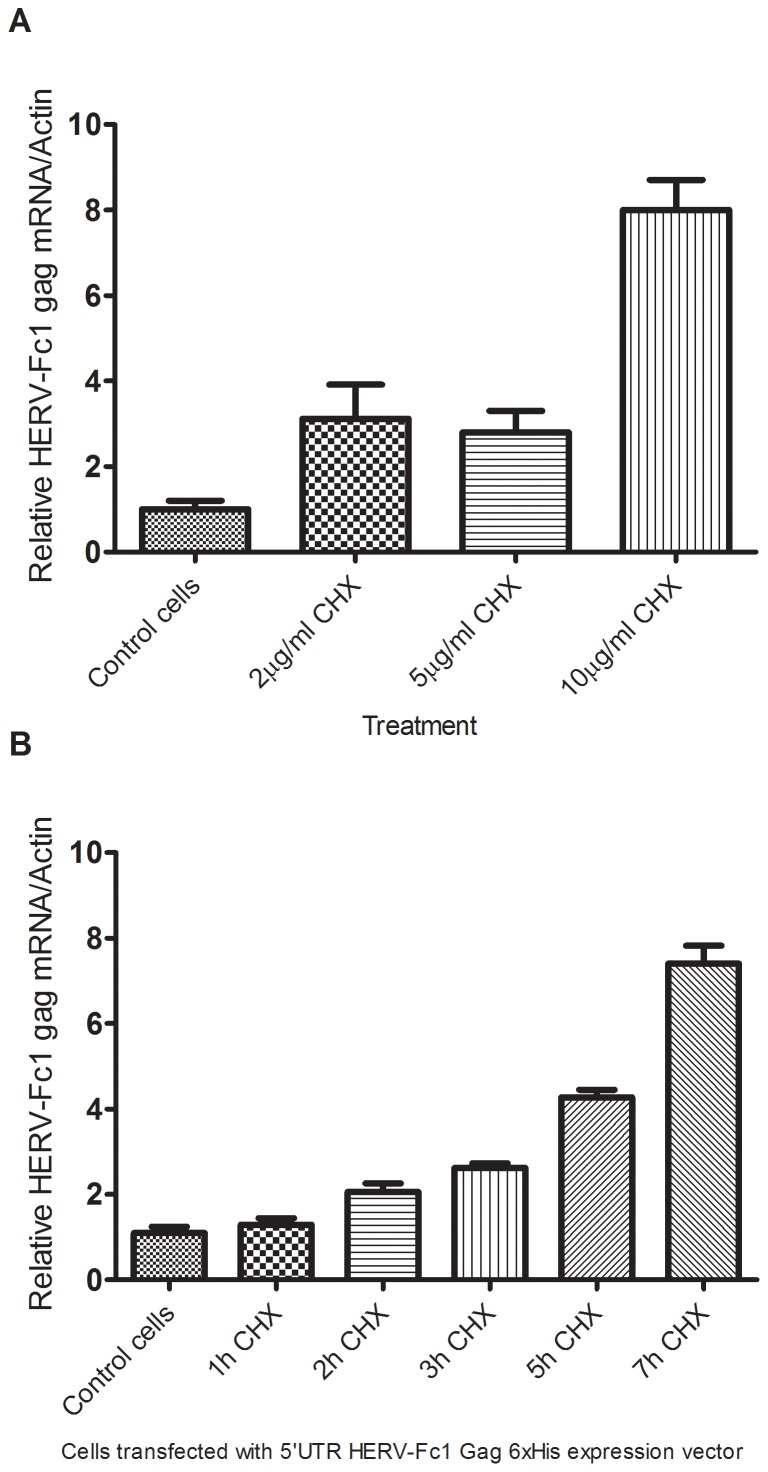
Cycloheximide increases endogenous HERV-Fc1 retroviral RNA levels in human cells. (A) Real-time Q-PCR analyses of the expression of HERV-Fc1 *gag* extracellular sequences in cells treated with increasing doses of cycloheximide. Graph bars represent relative changes of HERV-Fc1 *gag* extracellular mRNA. Q-PCR results shown are the means of two experiments each performed in triplicates. Errors bars represent ± SD. Y-axis relative HERV-Fc1 mRNA expression levels. (B) Time response curve of HERV-Fc1 mRNA induction in cells transfected with 5′UTR HERV-Fc1 Gag 6×His expression vector. Graph bars represent relative changes of HERV-Fc1 *gag* extracellular mRNA. Q-PCR results shown are the means of two experiments each performed in triplicates. Cycloheximide was added 48 h post transfection at the concentration of 10 µg/ml. Errors bars represent ± SD. Y-axis relative HERV-Fc1 mRNA expression levels.

**Figure 7:**
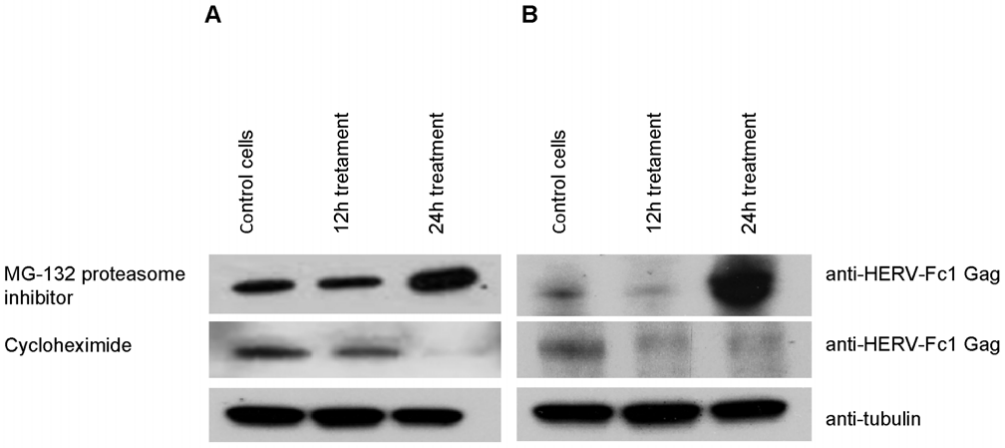
HERV-Fc1 Gag is subjected to proteasomal degradation. HEK 293 (A) and Jurkat cells (B) expressing exogenous HERV-Fc1 Gag protein were incubated, 16 hours post-transfection, with the translation inhibitor cycloheximide. After 12 h and 24 h, cells were collected and analyzed by Western blot using anti-HERV-Fc1 antibody. Cellular tubulin was analyzed as a loading control. HEK 293 (A) and Jurkat cells (B) expressing exogenous HERV-Fc1 Gag protein were incubated, 16 hours post-transfection, with proteasome inhibitor MG132 at 10 µM. Cells were collected every 12 h for, in total, 24 h and analyzed by Western blot using anti-HERV-Fc1 antibody.

Induction of HERV-Fc1 mRNA sequences was investigated as a function of cycloheximide concentration. Cells were exposed to different concentrations of this inhibitor of protein synthesis, ranging from 2–10 µg/ml and incubated for 12 h. Significant increase in the HERV-Fc1 *gag* mRNA induction was reached at dose of 10 µg/ml. The relative concentration of HERV-Fc1 *gag* RNA in cycloheximide-treated cells was increased 8-fold (Figure 6a). Thus our data reflects a quantitative increase in virus-specific sequences normally present in untreated cells. The time course of HERV-Fc1 *gag* mRNA virus activation in cells in response to cycloheximide was next investigated (Figure 6b). As shown in Figure 6b, maximal HERV-Fc1 *gag* mRNA levels produced by the cells were registered during the first 7 h after drug treatment. At later times showed a rapid decline in HERV-Fc1 *gag* mRNA activation (data not shown). Next, we assessed the intracellular HERV-Fc1 Gag protein decay in transfected cells. Figure 7a and b shows half-life of exogenously expressed HERV-Fc1 Gag proteins over 24 h following 10 µg/ml cycloheximide treatment started at 0 h. Cycloheximide was added to a Gag expressing cells 16 hours post-transfection. Controls were handled similarly, but with no cycloheximide added. Cells were lysed at the indicated time point and subjected to Western Blot analysis. HERV-Fc1 Gag protein levels decreased slowly but significantly after cycloheximide addition in HEK 293 and Jurkat cells by 24 h.

To investigate the molecular mechanisms responsible for HERV-Fc1 Gag degradation, proteasome inhibition was achieved by addition of carbobenzoxy-L-leucyl-L-leucyl-L-leucinal (MG132) to a final concentration of 10 µM. MG132 was added to Gag expressing HEK 293 or Jurkat cells 16 hours post-transfection. The cells were collected at the indicated time points and analyzed by Western Blot using the anti-HERV-Fc1 antibody. As shown in Figure 7a (HEK 293 cells) and 7b (Jurkat cells), for HERV-Fc1 Gag-transfected cells, expression of Gag was possible to detect at the start of the incubation. Treatment with MG132 for 12 h drastically increased the amount of Gag, and the Gag levels further increased over 24 h of incubation in presence of proteasome inhibitor. The HERV-Fc1 Gag protein levels increased by at least a factor of 10 in HEK 293 cells and 100 in Jurkat cells.

## Discussion

Transposable elements, including endogenous retroviruses (ERVs), are silenced by DNA methylation/chromatin structure in mammalian cells [30]. However, there have been very few experimental studies examining the methylation status of human ERVs. Chromatin status probably contributes to their regulation, and the CpG methylation status of HERV promoter and regulatory regions appears as a crucial factor for activity versus silencing [17] [30] [31]. Not much is known about transcriptional factors actually regulating transcriptional activity of individual HERV loci.

Several observations in mice [32] [33] and humans [34] suggest methylation to be a strong transcriptional repressor of transposed genes and could play a role in protecting the genome from the potentially harmful effects of inserted sequences as part of the genome defense system. LTR regulatory sequences are targets for repressing factors and *de novo* methylation of CpG islands. Methylated LTRs are inefficient as promoters, while unmethylated LTRs are functional. LTR methylation can be reversed by the demethylating agent 5-aza-dC, which is an irreversible inhibitor of cytosine 5-methyltransferases. Reactivation of retroelements might represent a particularly important consequence of DNA hypomethylation, since their repression is a key function of DNA methylation in mammalian genomes [9].

Cytosine methylation of CpG sites was proposed to cause retroviral silencing over 30 years ago. Chromosomally integrated adenovirus and herpes virus DNAs are much more methylated than their free virion DNAs. Investigations on mouse mammary tumor virus genome show that, in a genetically transmitted viral sequence, part of its genome is methylated, whereas the same sequence was devoid of DNA modification in the provirus acquired via milk-borne infection [35]. Furthermore regulation of type C virus gene expression at the transcriptional level has been reported for BALB: virus-1 and for ecotropic AKR virus [36]. Furthermore, present work demonstrates that CpG methylation of the HIV-1 5′LTR could be an important epigenetic mechanism that maintains the latency of HIV-1 proviruses by preventing their reactivation [37].

In this study, we determined and compared the methylation status of the 5′ long terminal repeats (LTRs) - *gag* region of the human endogenous retrovirus (HERV) HERV-Fc1 in a variety of human cells. Furthermore, we investigated factors regulating the expression of endogenous HERV-Fc1 *gag/env* extracellular RNA sequences and Gag proteins in those cells. In our present study, 5-aza-dC is shown to be a powerful inducer of HERV-Fc1, and at the same time it strongly inhibits methylation of DNA. Treatment with this demethylating agent 5-aza-dC, results in significantly increased levels of HERV-Fc1 expression in cells previously not expressing HERV-Fc1, or with a very low expression level. Three types of human cells were used in our studies, HEK 293 cells, Jurkat cells and primary PBMCs from healthy controls. Although there were quantitative differences, the fundamental patterns observed with the cells were similar: A 10 to 100-fold induction of the tested endogenous HERV-Fc1 retroviral elements. In one instance, expression of the HERV-Fc1 *gag* RNA in Jurkat cells, we quantified a 600-fold induction of the expression of HERV-Fc1 sequences. However, this strong induction was associated with a very low background level, rather than a very high induced level. Our results are in accordance with recently published data were up to 50 000 fold upregulation in HERV-Fc1 *env* expression was observed upon treatment of RL95-2 cell line with 5-aza-dC [38].

In our study, high levels of HERV-Fc1 transcription were the result of a disturbed genetic and epigenetic regulation of expression. DNA methylation was inhibited by 5-aza-dC at concentrations equivalent to those which induced viral RNA transcriptions and in one case HERV-Fc1 Gag protein expression. The present study indicates that the progeny of human cells that have been transiently exposed to 5-aza-dC display constitutive expression of transcripts related to HERV-Fc1 *gag* and/or *env* RNA sequences. We presume that this reflects demethylation of the related DNA sequences induced by the original exposure to 5-aza-dC. Indeed our subsequent methylation studies provided evidence for hypermethylation of the endogenous HERV-Fc1 5′LTR-*gag* DNA sequences in those cells. The extent of expression of HERV-Fc1 RNAs precisely correlates with the apparent extent of demethylation of the related DNA sequences. This is in accordance with previous evidences that the critical sites for the control of transcription by DNA methylation are confined to the 5′ end of genes [39]. In all cell lines studied, response to 5-aza-dC resulted in significant demethylation, and greater than 50% reduction was observed in all cells indicating significant DNA demethylation. Methylation levels of the examined HERV-Fc1 5′LTR-*gag* region vary between cell lines; however, in all of them it greatly surpassed 95%. We observed that transcriptional expression level of HERV-Fc1 RNA sequences negatively correlates with methylation levels.

Remethylation conversely decreased HERV-Fc1 *gag* RNA expression level. Hence, methylated CpG sites in HERV-Fc1 5′LTRs strongly inhibit transcriptional activity of HERV-Fc1 proviral loci. These findings further corroborate that CpG methylation plays an important and direct role in the transcriptional regulation of the HERV-Fc1. After 5-aza-dC treatment, when the cells were transferred to drug-free medium, demethylation was reversed and HERV-Fc1 expression was reduced. However, despite full remethylation of the LTR, the gene was still expressed at moderate levels, by the tenth day of drug free growth. Further studies on methylation levels in single clones derived from single cells after 5-aza-dC treatment are necessary to determine whether the remethylation observed resulted from *de novo* methylation of from the selection of cells in which CpG islands had not become demethylated by the drug. Consequently we speculate that 5-aza-dC induced expression of HERV-Fc1 can be driven from a largely methylated promoter possibly with localized demethylation at the transcription start side.

The mechanism by which 5-aza-dC inhibits DNA methylation is still unknown. Santi et al. have suggested that 5-aza-dC causes a mechanistic inhibition of the processive methylating enzymes and therefore, has an effect that exceeds the level of incorporation of the compound into the DNA [40]. With this kind of effect the enzyme stalls when it comes to a 5-aza-residue in the DNA and is unable to proceed. This leads to hypomethylation of normal cytosine residues that may be downstream from the site of enzyme stalling [8]. The incorporation of 5-aza-dC into DNA also increases the formation of stable, protein- DNA complexes with other nuclear proteins [41]. These tight-binding complexes may involve regulatory protein factors as well as enzymes associated with DNA. In that way, the perturbations of DNA-protein interactions caused by 5-aza-dC may interfere with normal cellular functioning and thus be responsible for some of the reflective changes in gene expression elicited by the drug.

Demethylation is obviously necessary for the increased expression of HERV-Fc1. In physiological conditions, it is unlikely that HERV-Fc1 is able to demethylate its provirus, the loss of methylation must be the result of genetic changes in the cell, e.g., a loss of DNMT1 activity. Our results indicate that 5-aza-dC and TSA mediated enhancement of transcription of HERV-Fc1 *gag* and *env* RNA sequences is related to decrease in expression of DNMT1. The mechanism whereby the DNMT1-DNA adducts induces DNMT1 degradation has not been elucidated in this study. As the induction of hypomethylation correlates better with the 5-aza-dC dose than the DNMT1 level our results agree with previous reports [42] that trapping of chromatin bound DNMT1 molecules by 5-aza-dC is the primary cause of hypomethylation.

Physiologically, lack of DNMT1 activity is probably involved in genome-wide demethylation occurring during normal embryonic development and cell differentiation [43]. A discrepancy between DNMT1 expression and cell proliferation is thought to be responsible for T-cell DNA hypomethylation in systemic lupus erythematous (SLE) [43]. 5-aza-dC increases HERV clone 4-1-like messenger RNA in healthy controls, but not in patients with SLE [44]. Furthermore, drugs like procainamide and hydralazine that induce a lupus-like illness in genetically predisposed individuals are known to act as demethylating agents [45]. These drugs may promote autoimmunity by facilitating the transcription of endogenous viral antigens such as HERV antigens.

Also the role of histone acetylation in the transcription of HERV-Fc1 genes has been investigated here. We used an experimental system, in which cellular histone acetylation levels were shifted towards the hyperacetylated state. This was achieved using the broad HDAC inhibitor TSA. TSA treatment led to a global hyperacetylation of histone H3 and H4. TSA treatment in PBMCs strongly enhanced expression of HERV-Fc1 *gag* and *env* related transcripts. Our results indicate that in primary cells TSA is able to reactivate endogenous HERV-Fc1 sequences, more efficiently and with quicker kinetics (data not shown) than 5-aza-dC. Treatment with both drugs was not synergistic in regulating HERV-Fc1 expression in terms of extension and progressive upregulation.

Our results with PBMC cells are consistent with previous findings, in which TSA alone was found to reactivate methylated genes [46]. They also highlight the variability in epigenetic regulation between primary cells and cell lines, providing valuable data for further functional investigation in different cell populations. It has been reported that mammalian cells grown in culture exhibit differences in DNA methylation compared with normal uncultured cells. Primary cultures approaching senescence demonstrate decreasing DNMT1 activity and decreased 5-methylcytosine (5 mC) content, while immortalized cultures do not [47]. Recently Oka et al. suggested that the effect of 5-aza-dC is mediated primarily through DNMT3 and DNMT3b, thus differences in DNMT3 expression between different cell lines could account for altered sensitivity to the 5-aza-dC treatments. It was also suggested that the drug transport property of different cells may be a cause for the differential sensitivities [48].

The increased levels of HERV-Fc1 *gag* and/or *env* RNAs could reflect increased de novo transcription, decreased turnover of the related RNAs, or both. In our initial screen for drugs that would reactivate expression of HERV-Fc1, we likewise included protein synthesis inhibitor cycloheximide. With respect to the effects of cycloheximide, it is of interest that protein synthesis inhibitors can induce expression of the viral *gag* gene at the level of transcription. Our results confirm a hypothesis according to which cycloheximide causes decay of a labile control protein that either inhibits viral RNA transcription or acts at a post-transcriptional level to degrade viral RNA [49].

Taken together, DNA methylation might be only one of several independent silencing pathways.

Sequence determinants and mechanisms involved in LTR methylation need to be further investigated to better understand HERV regulations and deregulations associated with pathologies. The cellular (transcription) factors contributing to high HERV-Fc1 expression remain to be elucidated in future studies.

## Supporting Information

Figure S1 **HERV-Fc1 Gag protein synthesis in 5-aza-dC treated cells.** Western blotting for HERV-Fc1 Gag protein after 5-aza-dC, TSA or 5-aza-dC and TSA treatments in HEK 293 cells. No detectable endogenous HERV-Fc1 Gag protein in HEK 293 cells before drug treatment. Drug combinations and concentrations are indicated above gel lanes. Cells transfected with pcDNA3.1 (+)/mycHis A Fc1 Gag expression vector serve as a positive control. Result shown is a representative of two independent experiments performed on different protein extracts. Anti-tubulin antibody was used as a loading control.
(TIF)

Figure S2 **Effect of TSA dose course of treatment on acetylated histones.** Jurkat cells were treated with increasing doses of TSA. Cells were harvested, collected and analyzed by Western blotting using anti-acetyl histone H3 and H4 antibodies. Cellular tubulin was analyzed as a loading control.
(TIF)

## References

[1] Bird AP, Wolffe AP (1999) Methylation-induced repression–belts, braces, and chromatin. Cell 99: 451–454. 10589672 10.1016/s0092-8674(00)81532-9

[2] Jaenisch R, Bird A (2003) Epigenetic regulation of gene expression: how the genome integrates intrinsic and environmental signals. Nat Genet 33 Suppl: 245–254. 12610534 10.1038/ng1089

[3] Venter JC, Adams MD, Myers EW, Li PW, Mural RJ, et al (2001) The sequence of the human genome. Science 291: 1304–1351. 11181995 10.1126/science.1058040

[4] Miller OJ, Schnedl W, Allen J, Erlanger BF (1974) 5-Methylcytosine localised in mammalian constitutive heterochromatin. Nature 251: 636–637. 4609195 10.1038/251636a0

[5] Weber M, Schubeler D (2007) Genomic patterns of DNA methylation: targets and function of an epigenetic mark. Curr Opin Cell Biol 19: 273–280. 17466503 10.1016/j.ceb.2007.04.011

[6] Attwood JT, Yung RL, Richardson BC (2002) DNA methylation and the regulation of gene transcription. Cell Mol Life Sci 59: 241–257. 11915942 10.1007/s00018-002-8420-z PMC11146104

[7] Klose RJ, Bird AP (2006) Genomic DNA methylation: the mark and its mediators. Trends Biochem Sci 31: 89–97. 16403636 10.1016/j.tibs.2005.12.008

[8] Rascati RJ (1988) Effects of cytidine analogs on methylation of DNA and retrovirus induction. Mutat Res 208: 21–25. 3259285 10.1016/0165-7992(88)90015-2

[9] Yoder JA, Walsh CP, Bestor TH (1997) Cytosine methylation and the ecology of intragenomic parasites. Trends Genet 13: 335–340. 9260521 10.1016/s0168-9525(97)01181-5

[10] Bannert N, Kurth R (2004) Retroelements and the human genome: new perspectives on an old relation. Proc Natl Acad Sci U S A 101 Suppl 2: 14572–14579. 15310846 10.1073/pnas.0404838101 PMC521986

[11] Ogasawara H, Okada M, Kaneko H, Hishikawa T, Sekigawa I, et al (2003) Possible role of DNA hypomethylation in the induction of SLE: relationship to the transcription of human endogenous retroviruses. Clin Exp Rheumatol 21: 733–738. 14740452

[12] Johnston JB, Silva C, Holden J, Warren KG, Clark AW, et al (2001) Monocyte activation and differentiation augment human endogenous retrovirus expression: implications for inflammatory brain diseases. Ann Neurol 50: 434–442. 11601494 10.1002/ana.1131

[13] Wentzensen N, Coy JF, Knaebel HP, Linnebacher M, Wilz B, et al (2007) Expression of an endogenous retroviral sequence from the HERV-H group in gastrointestinal cancers. Int J Cancer 121: 1417–1423. 17546591 10.1002/ijc.22826

[14] Florl AR, Lower R, Schmitz-Drager BJ, Schulz WA (1999) DNA methylation and expression of LINE-1 and HERV-K provirus sequences in urothelial and renal cell carcinomas. Br J Cancer 80: 1312–1321. 10424731 10.1038/sj.bjc.6690524 PMC2363067

[15] Menendez L, Benigno BB, McDonald JF (2004) L1 and HERV-W retrotransposons are hypomethylated in human ovarian carcinomas. Mol Cancer 3: 12. 15109395 10.1186/1476-4598-3-12 PMC411053

[16] Sekigawa I, Ogasawara H, Kaneko H, Hishikawa T, Maruyama N (1997) [Systemic lupus erythematosus (SLE) and retrovirus]. Nihon Rinsho 55: 1492–1497. 9200938

[17] Lavie L, Kitova M, Maldener E, Meese E, Mayer J (2005) CpG methylation directly regulates transcriptional activity of the human endogenous retrovirus family HERV-K(HML-2). J Virol 79: 876–883. 15613316 10.1128/JVI.79.2.876-883.2005 PMC538560

[18] Benit L, Calteau A, Heidmann T (2003) Characterization of the low-copy HERV-Fc family: evidence for recent integrations in primates of elements with coding envelope genes. Virology 312: 159–168. 12890629 10.1016/s0042-6822(03)00163-6

[19] Jern P, Sperber GO, Blomberg J (2004) Definition and variation of human endogenous retrovirus H. Virology. 327: 93–110. 10.1016/j.virol.2004.06.023 15327901

[20] Kjellman C, Sjogren HO, Widegren B (1999) HERV-F, a new group of human endogenous retrovirus sequences. J Gen Virol 80 Pt 9: 2383–2392. 10501491 10.1099/0022-1317-80-9-2383

[21] Patzke S, Lindeskog M, Munthe E, Aasheim HC (2002) Characterization of a novel human endogenous retrovirus, HERV-H/F, expressed in human leukemia cell lines. Virology 303: 164–173. 12482668 10.1006/viro.2002.1615

[22] Nexo BA, Christensen T, Frederiksen J, Moller-Larsen A, Oturai AB, et al (2011) The etiology of multiple sclerosis: genetic evidence for the involvement of the human endogenous retrovirus HERV-Fc1. PLoS One 6: e16652. 21311761 10.1371/journal.pone.0016652 PMC3032779

[23] Laska MJ, Brudek T, Nissen KK, Christensen T, Moller-Larsen A, et al (2012) Expression of HERV-Fc1, a human endogenous retrovirus, is increased in patients with active multiple sclerosis. J Virol 86: 3713–3722. 22278236 10.1128/JVI.06723-11 PMC3302483

[24] Cameron EE, Bachman KE, Myohanen S, Herman JG, Baylin SB (1999) Synergy of demethylation and histone deacetylase inhibition in the re-expression of genes silenced in cancer. Nat Genet 21: 103–107. 9916800 10.1038/5047

[25] Stengel S, Fiebig U, Kurth R, Denner J (2010) Regulation of human endogenous retrovirus-K expression in melanomas by CpG methylation. Genes Chromosomes Cancer 49: 401–411. 20095041 10.1002/gcc.20751

[26] Bartoli A, Fettucciari K, Fetriconi I, Rosati E, Di Ianni M, et al (2003) Effect of trichostatin a and 5′-azacytidine on transgene reactivation in U937 transduced cells. Pharmacol Res 48: 111–118. 12770523

[27] Juttermann R, Li E, Jaenisch R (1994) Toxicity of 5-aza-2′-deoxycytidine to mammalian cells is mediated primarily by covalent trapping of DNA methyltransferase rather than DNA demethylation. Proc Natl Acad Sci U S A 91: 11797–11801. 7527544 10.1073/pnas.91.25.11797 PMC45322

[28] McInerney JM, Nawrocki JR, Lowrey CH (2000) Long-term silencing of retroviral vectors is resistant to reversal by trichostatin A and 5-azacytidine. Gene Ther 7: 653–663. 10800088 10.1038/sj.gt.3301155

[29] Schermelleh L, Spada F, Easwaran HP, Zolghadr K, Margot JB, et al (2005) Trapped in action: direct visualization of DNA methyltransferase activity in living cells. Nat Methods 2: 751–756. 16179921 10.1038/nmeth794

[30] Reiss D, Zhang Y, Mager DL (2007) Widely variable endogenous retroviral methylation levels in human placenta. Nucleic Acids Res 35: 4743–4754. 17617638 10.1093/nar/gkm455 PMC1950553

[31] Matouskova M, Blazkova J, Pajer P, Pavlicek A, Hejnar J (2006) CpG methylation suppresses transcriptional activity of human syncytin-1 in non-placental tissues. Exp Cell Res 312: 1011–1020. 16427621 10.1016/j.yexcr.2005.12.010

[32] Barlow DP (1993) Methylation and imprinting: from host defense to gene regulation? Science 260: 309–310. 8469984 10.1126/science.8469984

[33] Duhl DM, Vrieling H, Miller KA, Wolff GL, Barsh GS (1994) Neomorphic agouti mutations in obese yellow mice. Nat Genet 8: 59–65. 7987393 10.1038/ng0994-59

[34] Liu WM, Schmid CW (1993) Proposed roles for DNA methylation in Alu transcriptional repression and mutational inactivation. Nucleic Acids Res 21: 1351–1359. 8464725 10.1093/nar/21.6.1351 PMC309319

[35] Sutter D, Doerfler W (1980) Methylation of integrated adenovirus type 12 DNA sequences in transformed cells is inversely correlated with viral gene expression. Proc Natl Acad Sci U S A 77: 253–256. 6244548 10.1073/pnas.77.1.253 PMC348247

[36] Niwa O, Sugahara T (1981) 5-Azacytidine induction of mouse endogenous type C virus and suppression of DNA methylation. Proc Natl Acad Sci U S A 78: 6290–6294. 6171816 10.1073/pnas.78.10.6290 PMC349024

[37] Blazkova J, Trejbalova K, Gondois-Rey F, Halfon P, Philibert P, et al (2009) CpG methylation controls reactivation of HIV from latency. PLoS Pathog 5: e1000554. 19696893 10.1371/journal.ppat.1000554 PMC2722084

[38] Strissel PL, Ruebner M, Thiel F, Wachter D, Ekici AB, et al (2012) Reactivation of codogenic endogenous retroviral (ERV) envelope genes in human endometrial carcinoma and prestages: Emergence of new molecular targets. Oncotarget 10.18632/oncotarget.679 PMC3717959 23085571

[39] Brenet F, Moh M, Funk P, Feierstein E, Viale AJ, et al (2011) DNA methylation of the first exon is tightly linked to transcriptional silencing. PLoS One 6: e14524. 21267076 10.1371/journal.pone.0014524 PMC3022582

[40] Santi DV, Garrett CE, Barr PJ (1983) On the mechanism of inhibition of DNA-cytosine methyltransferases by cytosine analogs. Cell 33: 9–10. 6205762 10.1016/0092-8674(83)90327-6

[41] Michalowsky LA, Jones PA (1987) Differential nuclear protein binding to 5-azacytosine-containing DNA as a potential mechanism for 5-aza-2′-deoxycytidine resistance. Mol Cell Biol 7: 3076–3083. 2444874 10.1128/mcb.7.9.3076-3083.1987 PMC367939

[42] Patel K, Dickson J, Din S, Macleod K, Jodrell D, et al (2010) Targeting of 5-aza-2′-deoxycytidine residues by chromatin-associated DNMT1 induces proteasomal degradation of the free enzyme. Nucleic Acids Res 38: 4313–4324. 20348135 10.1093/nar/gkq187 PMC2910061

[43] Rougier N, Bourc'his D, Gomes DM, Niveleau A, Plachot M, et al (1998) Chromosome methylation patterns during mammalian preimplantation development. Genes Dev 12: 2108–2113. 9679055 10.1101/gad.12.14.2108 PMC317005

[44] Okada M, Ogasawara H, Kaneko H, Hishikawa T, Sekigawa I, et al (2002) Role of DNA methylation in transcription of human endogenous retrovirus in the pathogenesis of systemic lupus erythematosus. J Rheumatol 29: 1678–1682. 12180729

[45] Yung RL, Quddus J, Chrisp CE, Johnson KJ, Richardson BC (1995) Mechanism of drug-induced lupus. I. Cloned Th2 cells modified with DNA methylation inhibitors in vitro cause autoimmunity in vivo. J Immunol 154: 3025–3035. 7533191

[46] Courtier B, Heard E, Avner P (1995) Xce haplotypes show modified methylation in a region of the active X chromosome lying 3′ to Xist. Proc Natl Acad Sci U S A 92: 3531–3535. 7536936 10.1073/pnas.92.8.3531 PMC42201

[47] Vertino PM, Issa JP, Pereira-Smith OM, Baylin SB (1994) Stabilization of DNA methyltransferase levels and CpG island hypermethylation precede SV40-induced immortalization of human fibroblasts. Cell Growth Differ 5: 1395–1402. 7696189

[48] Oka M, Meacham AM, Hamazaki T, Rodic N, Chang LJ, et al (2005) De novo DNA methyltransferases Dnmt3a and Dnmt3b primarily mediate the cytotoxic effect of 5-aza-2′-deoxycytidine. Oncogene 24: 3091–3099. 15735669 10.1038/sj.onc.1208540

[49] Aaronson SA, Anderson GR, Dunn CY, Robbins KC (1974) Induction of type-C RNA virus by cycloheximide: increased expression of virus-specific RNA. Proc Natl Acad Sci U S A 71: 3941–3945. 4372598 10.1073/pnas.71.10.3941 PMC434302

