# Supplementary figures and images for: (Some) Cellular Mechanisms Influencing the Transcription of Human Endogenous Retrovirus, HERV-Fc1
Source: PLoS One. 2013 Jan 28;8(1):e53895. doi: 10.1371/journal.pone.0053895 (PMC3557288; doi:10.1371/journal.pone.0053895)

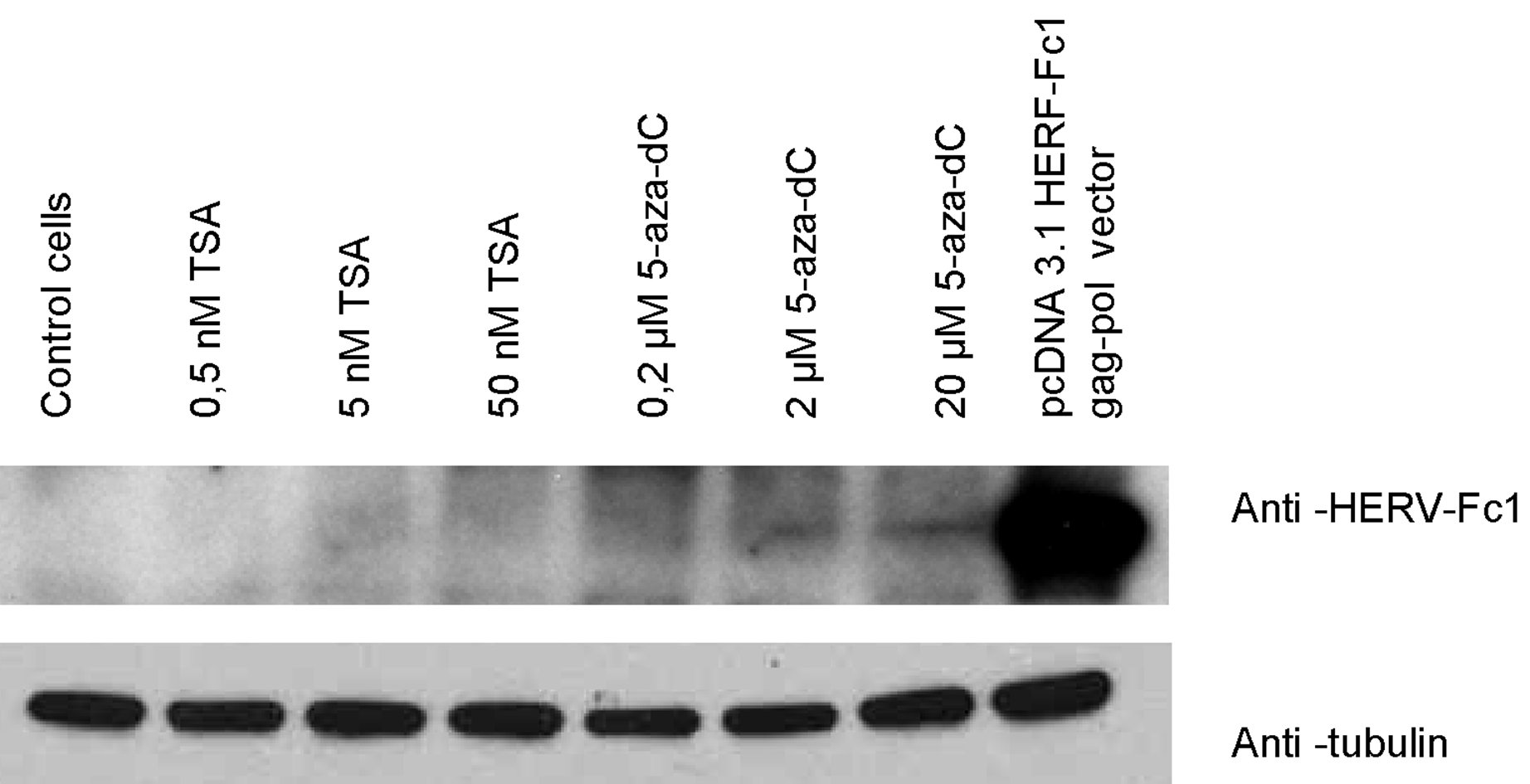

Supplement: Figure S1 — HERV-Fc1 Gag protein synthesis in 5-aza-dC treated cells. Western blotting for HERV-Fc1 Gag protein after 5-aza-dC, TSA or 5-aza-dC and TSA treatments in HEK 293 cells. No detectable endogenous HERV-Fc1 Gag protein in HEK 293 cells before drug treatment. Drug combinations and concentrations are indicated above gel lanes. Cells transfected with pcDNA3.1 (+)/mycHis A Fc1 Gag expression vector serve as a positive control. Result shown is a representative of two independent experiments performed on different protein extracts. Anti-tubulin antibody was used as a loading control. (TIF) [file pone.0053895.s001.tif]

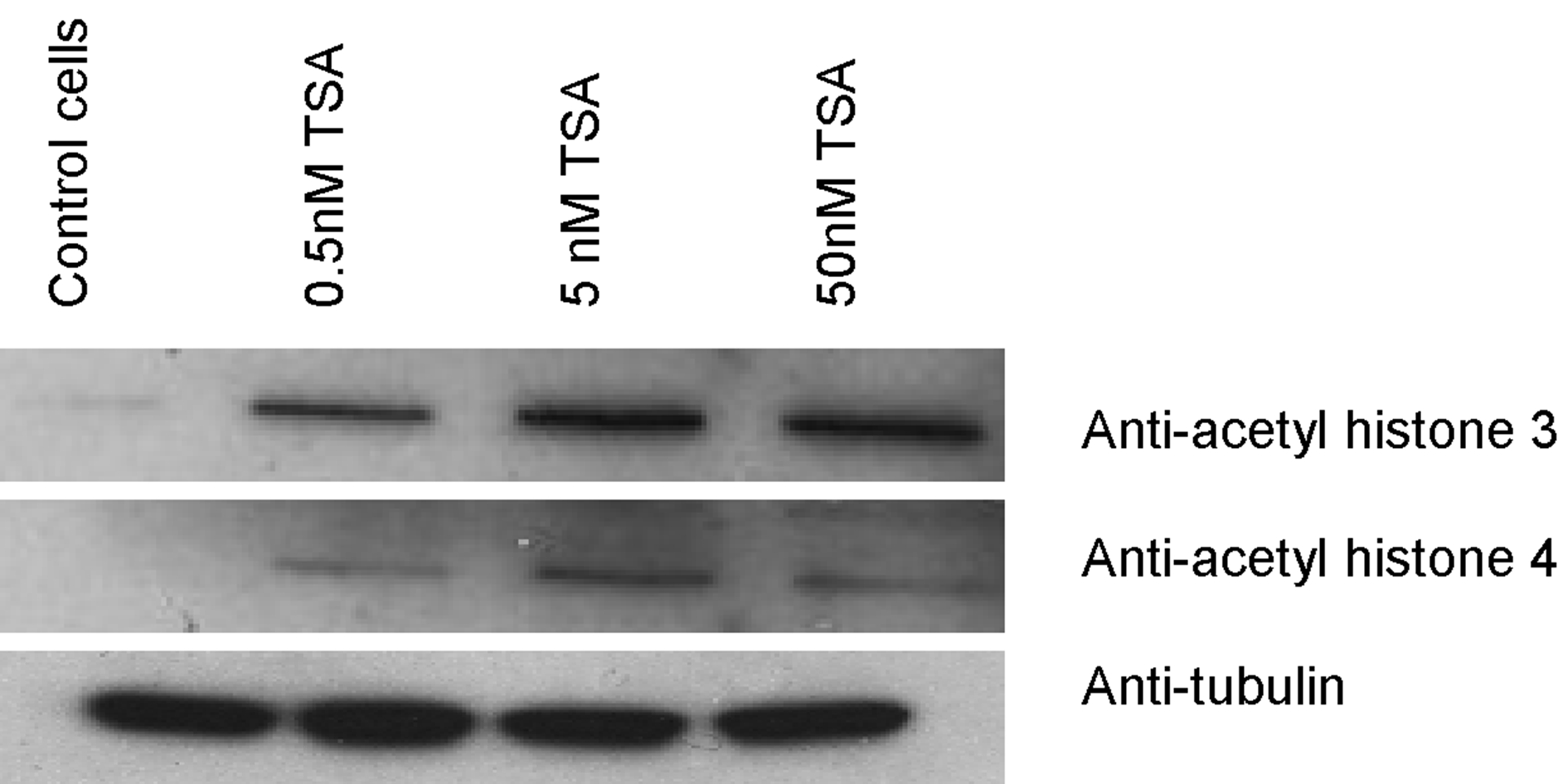

Supplement: Figure S2 — Effect of TSA dose course of treatment on acetylated histones. Jurkat cells were treated with increasing doses of TSA. Cells were harvested, collected and analyzed by Western blotting using anti-acetyl histone H3 and H4 antibodies. Cellular tubulin was analyzed as a loading control. (TIF) [file pone.0053895.s002.tif]
